# An experimental study on the thermal characteristics and heating effect of arc-fault from Cu core in residential electrical wiring fires

**DOI:** 10.1371/journal.pone.0182811

**Published:** 2017-08-10

**Authors:** Jian-Hua Du, Ran Tu, Yi Zeng, Leng Pan, Ren-Cheng Zhang

**Affiliations:** 1 College of Mechanical Engineering and Automation, Huaqiao University, Xiamen, Fujian, China; 2 College of Tourism, Huaqiao University, Quanzhou, Fujian, China; Universite de Lorraine, FRANCE

## Abstract

The characteristics of a series direct current (DC) arc-fault including both electrical and thermal parameters were investigated based on an arc-fault simulator to provide references for multi-parameter electrical fire detection method. Tests on arc fault behavior with three different initial circuit voltages, resistances and arc gaps were conducted, respectively. The influences of circuit conditions on arc dynamic image, voltage, current or power were interpreted. Also, the temperature rises of electrode surface and ambient air were studied. The results showed that, first, significant variations of arc structure and light emitting were observed under different conditions. A thin outer burning layer of vapor generated from electrodes with orange light was found due to the extremely high arc temperature. Second, with the increasing electrode gap in discharging, the arc power was shown to have a non monotonic relationship with arc length for constant initial circuit voltage and resistance. Finally, the temperature rises of electrode surface caused by heat transfer from arc were found to be not sensitive with increasing arc length due to special heat transfer mechanism. In addition, temperature of ambient air showed a large gradient in radial direction of arc.

## Introduction

Arc-fault is a kind of unintentional arcing condition occurred in electrical circuits, which is believed to be caused by loose connection, insulation failure or circuit aging, etc [1–5]. As the continuous heat release and luminous discharge of electricity, arc-fault is a potential inducement of fires. In 2006, an official report from United States Fire Administration (USFA) indicated that arc-fault had already become one of the major reasons for electrical fires in America [6]. Until now arc-fault is not only an issue of home fire safety, but also a huge threat to electrical appliances used in special fields for public such as power station, air and space craft [7–8].

Fires caused by arc-fault usually started from the ignition of combustible materials nearby, especially the insulating material of electrical wire made by e.g., polyethylene (PE), polyvinyl chloride (PVC) or acrylonitrile butadiene styrene (ABS). When an arc forms, the heat is not localized at the fault point but transferred to the entire circuit through inner metal core and insulation of electrical wire [9–11]. If the insulation has been preheated to more than 300°C as a result of heat accumulation from arc or Joule heating, the ignition can even occur to produce a flame [9].

To prevent electrical fires from happening, many previous studies focused on the detection method of arc-fault. Considering the form of arc, an arc-fault could be either a series arc-fault or a parallel arc-fault generally [3], on the other hand could be a direct current (DC) arc-fault or an alternating current (AC) arc-fault, which are very different in electrical characteristics. By monitoring the circuit state in timely manner, arc-fault circuit interrupter (AFCI) technology and arc-fault detectors (AFD) were developed e.g. in [4, 8, 12–15], and have been proved to be effective to prevent potential electrical fire. However, as most of the present arc detection methods and criterions were based on the variation of circuit electrical parameters (which are affected by types of arc forms, circuit loads etc), it’s still difficult to provide a general detection method suitable for all kinds of arc-faults to avoid false alarm or fail to alarm completely.

Expect for electrical characteristics, thermal characteristics including e.g., temperature, radiation and thermography, were thought to be another important category of parameters for electrical fire detection [16–18]. As the enhancement of heat transfer by arc, the developing behavior of fire induced by arc-fault is relatively special comparing to other fires. It could be expected that detection combing the electrical and thermal characteristics would be a more effective method for arc-fault detection.

In this study, the development of electrical and thermal characteristics of arc-fault at the early stage was investigated using an arc-fault simulator. Series DC arc-faults with different shapes and intensity were chosen for good stability and repeatability. Phenomenological analyses on arc images, variations of arc current and voltage, temperature rises of electrode and ambient air from experimental data were provided to give an inspiration for the prevention or early detection of electrical fire by arc-fault.

## Methods

### Arc-fault simulator platform

**Fig 1:**
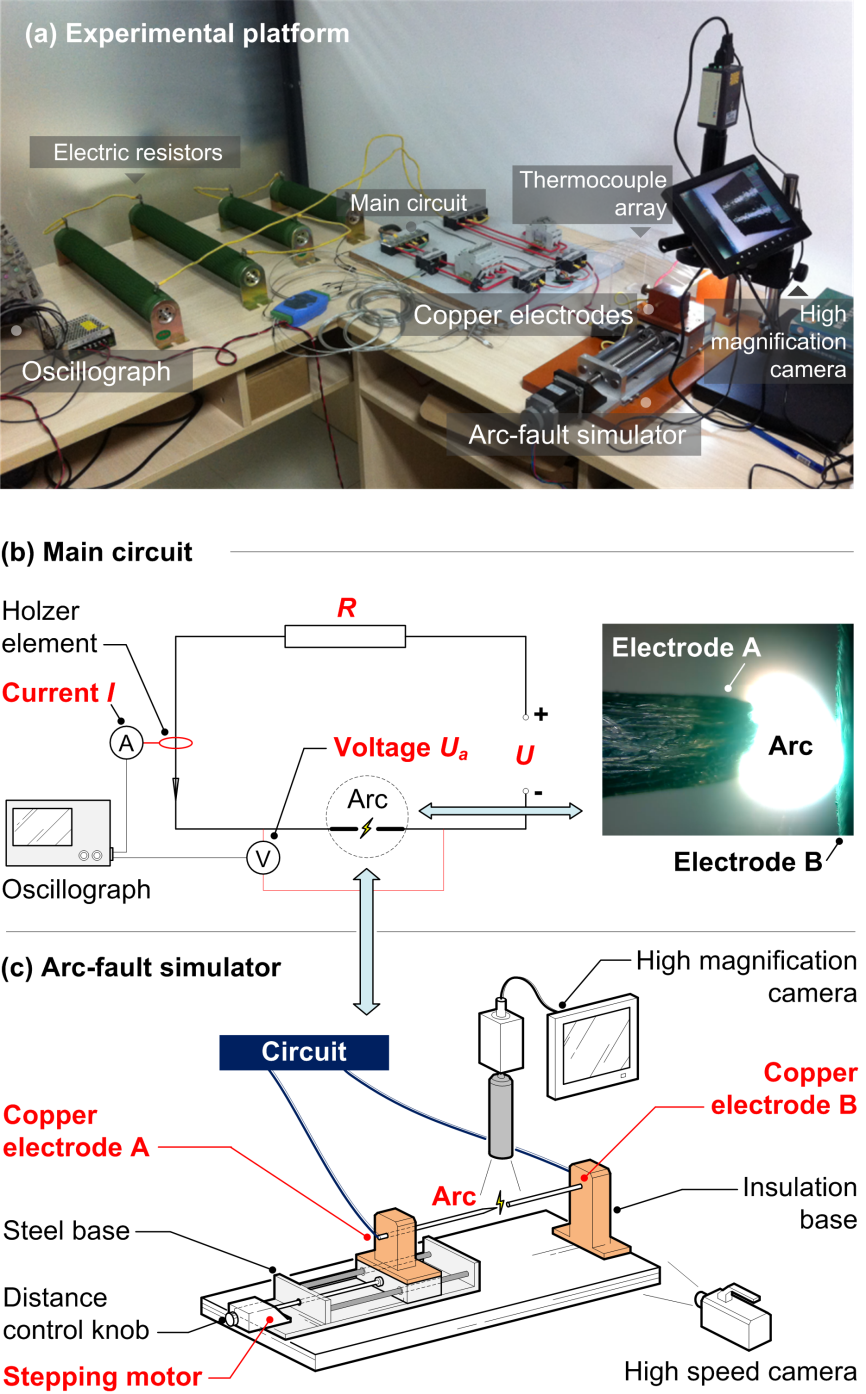
Experimental setup (a) and sketch of main circuit (b) with arc-fault simulator (c).

Experiments on the thermal behavior of arc-fault were conducted using a DC arc-fault simulator as shown in Fig 1. The main circuit, consists of a DC power supply, resistor array and an arc-fault simulator, is shown in Fig 1(B). The DC power supply (ETS-1000X10 from Ametek, America) could provide a variable DC voltage *U* with a maximum 1000 V and uncertainty within 0.2%. The resistor array was made of several high power corrugated resistors (with electric resistance 20 Ω and maximum power 2000 W, from GEE Electronics, China) by series connection. The total resistance *R* could be altered by changing the number of corrugated resistors (e.g., *R* was adjusted to be 40 Ω, 60 Ω and 80 Ω during tests, respectively). A Hall effect current sensor (CHB25-NP from Sensor Electronics, China) was used to measure the current *I* variation in circuit with a maximum 25 A and uncertainty within 0.8%. The current *I* and arc voltage *U*_*a*_ were both recorded by an oscillograph (DPO4010B-L from Tektronix, America) online. In order to obtain high quality data, the sampling frequency was chosen to be 100 Hz.

A more detailed diagram of the arc-fault simulator is shown in Fig 1(C). This simulator was designed based on standard UL1699B [19]. With the proper terminal voltage and electrode gap, the arc could be generated between a couple of coaxial copper electrodes A (Anode) and B (Cathode), which were mounted in insulation bases respectively. The discharge tips of electrode A and B are designed to be cone shape and flat shape respectively in the tests. Considering the electrode gap should be adjustable, the insulation base of electrode A was fixed on a steel sliding block, which could only make a one dimensional movement along the direction parallel to the two electrodes. The movement velocity and distance of electrode A from B, i.e., the length of electrode gap (or arc gap when discharging) *L*, was controlled by a programmable stepping motor, which could lead the sliding block move forward or backward precisely according to a screw structure rod. Also, there is another way to give a fine tuning to the gap *L* by hand using the distance control knob behind the stepping motor.

**Fig 2:**
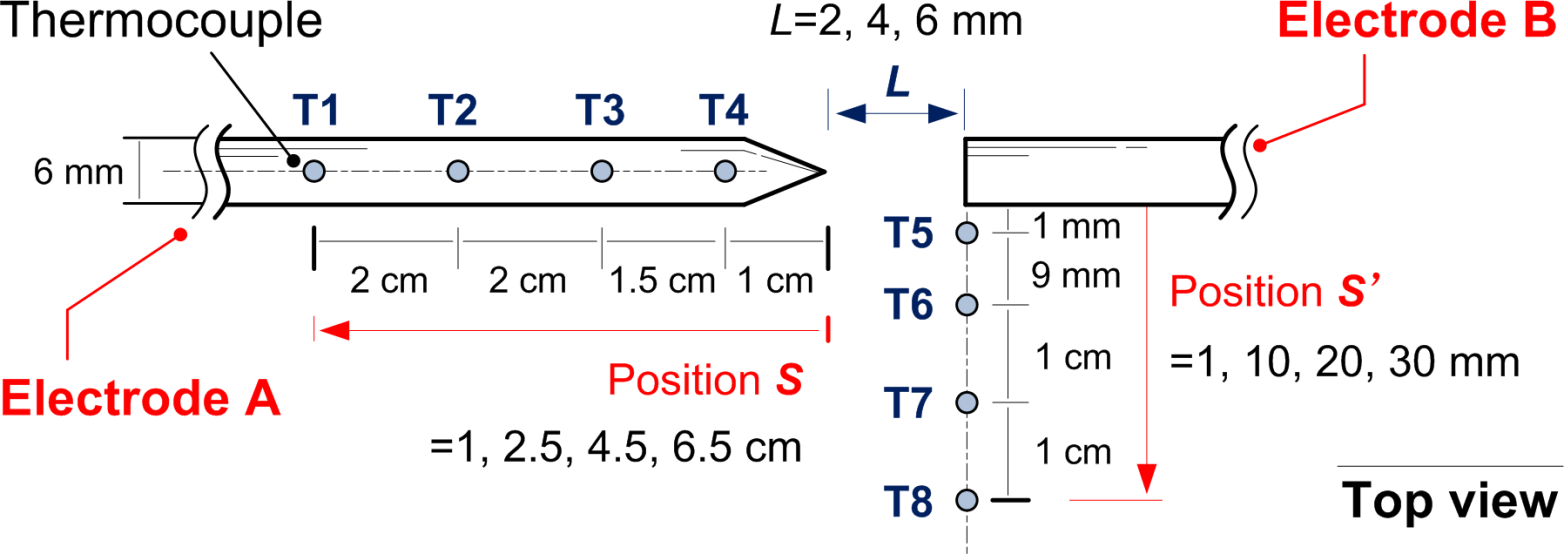
Detailed thermocouple array arrangement for temperature measurement.

For a better observation of the arc with small scale, a high magnification digital camera (TD-208A from Taida Instruments, China) with 30 frames per second and maximum magnification rate 620 was set right above the electrode gap to record the arc shape from top view. Another use of this camera was to confirm or assist correcting the gap distance controlled by the stepping motor. A high speed camera (Phantom Miro M110 from Vision Research, America) with maximum 1630 frames per second was used to monitor the arc burning behavior from side view. As shown in Fig 2, temperatures were measured by thermocouple array (T1-T8) with each diameter 0.5 mm and uncertainty within 0.75%. T1-T4 were fixed on the surface of electrode A, which showed the surface temperature with position *S* (distance from the tip of electrode A) 1 cm, 2.5 cm, 4.5 cm and 6.5 cm. T5-T8 showed the ambient air temperature rises by arc heating with position *S*’ (distance from the edge of electrode B) 1mm, 1cm, 2cm and 3cm, respectively. Any trade name mentioned above is only for descriptive purpose.

### Experimental procedure

The influences of circuit voltage, resistance and arc gap on arc characteristic behavior were considered in the present experiments. As summarized in Table 1, tests were designed to study the arc behavior with different intensity or shape. Electrodes A and B were shorted together at the beginning of each test. When the circuit was energized, electrode A would then be moved backward by the stepping motor in exact 8 seconds to obtain the preset arc gap. The duration of each test or arc discharging time was set to be 50 seconds to prevent the over-heating and melting of electrodes by high temperature plasma stream of arc. Compared to a very limited test time in standard UL1699B, a relatively “long” discharging time was used here to reflect the heat transfer process through arc-fault. Electrode A would be moved forward quickly to reconnect electrode B after that to extinguish the arc (test stopped). Because of high temperature from arc, some black residue was observed on electrode surface with the experimental process attributed to the oxidation of electrode material, which would be cleaned out by abrasive paper before each test. In addition, all the tests were repeated to ensure reproducible results within permitted error range.

**Table 1:** Experimental design of initial operating conditions.

Parameters	Preset value
Circuit voltage *U* /V	*U* = 100, 150, 200
Resistance *R* /Ω	*R* = 40, 60, 80 Ω
Arc gap *L* /mm	*L* = 2, 4, 6
Environmental	Ambient temperature 25 ± 2°C
	Humidity 50 ± 5%

## Results and discussion

### Arc image characteristics

**Fig 3:**
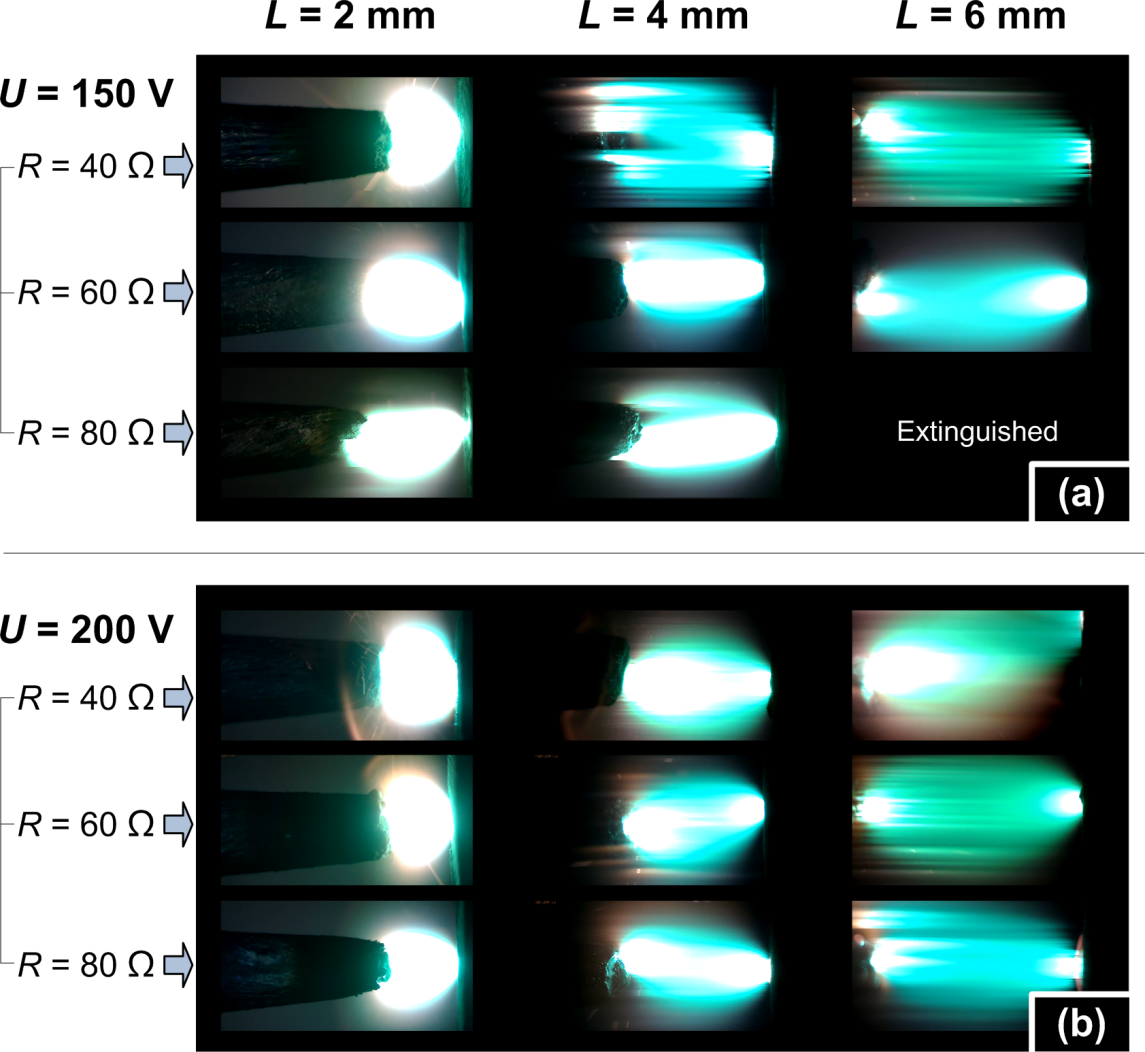
Top view of typical arc images with increasing arc gap and initial circuit voltage 150 V (a), 200 V (b).

As important visual features, arc dynamic image characteristics including color, geometry, flickering and so on could provide meaningful information for video-based fire detection technique. The typical arc images (top view by high magnification digital camera) with electrode gaps *L* = 2, 4, 6 mm under circuit *U* = 150, 200 V and *R* = 40, 60, 80 Ω are shown in Fig 3. For the circuit condition of *U* = 150 V, *R* = 80 Ω and *L* = 6 mm, the arc extinguished during growth, as a lower limit of arc voltage or current was proved to exist to maintain the plasma stream. With the increasing arc gaps, the arc light around core zone turned from white into blue attributed to the emitting of high temperature arc plasma flow. A luminous flame-like zone, emitting orange light, formed at the outer layer. This layer was believed to be a reaction zone for metal vapor and impurity substances generated from electrode by high arc temperature, and was more obvious at the higher voltage as Fig 3(B) shows. On the contrary, the influence of circuit voltage on the shape of arc core zone seemed not evident by Fig 3(A) and 3(B).

**Fig 4:**
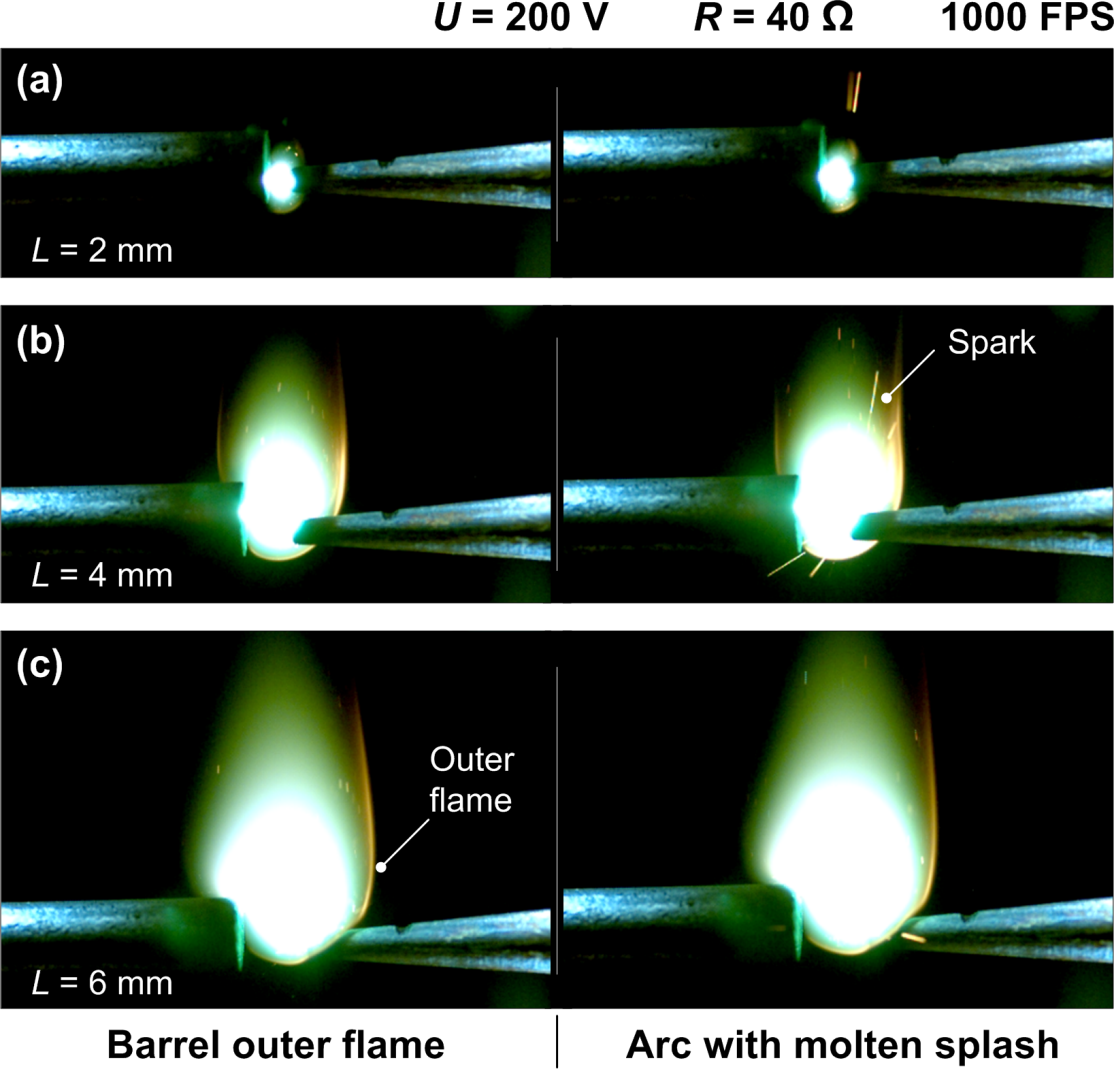
Front view of arc shape (left side) and spark phenomenon (right side) with arc gaps 2 mm (a), 4 mm (b) and 6 mm (c) under voltage 200 V and resistance 40 Ω.

As the arc stream flickered quickly, the high speed camera was employed for an in-depth observation. Fig 4 gives the high speed images from front view of arcs with gaps *L* = 2, 4, 6 mm and circuit condition of *U* = 200 V, *R* = 40 Ω. By the buoyancy effect of ambient air entrainment induced by the hot arc, the arc didn’t show a regular cylinder shape from front view but more like an arched shape. The barrel shape plasma with orange light at outer layer was very clear and increasing with enlarged arc gap. The right sides of Fig 4(A)–4(C) indicate a special phenomenon in arc combustion, i.e., the metal spatter caused by break of the molten metal bubble. Considering the melting point of electrode material Cu is ~1356 K, it is not surprising for the metal spatter because that the arc temperature is usually supposed to be far more than 5000 K [3].

### Variation of arc characteristic parameters

**Fig 5:**
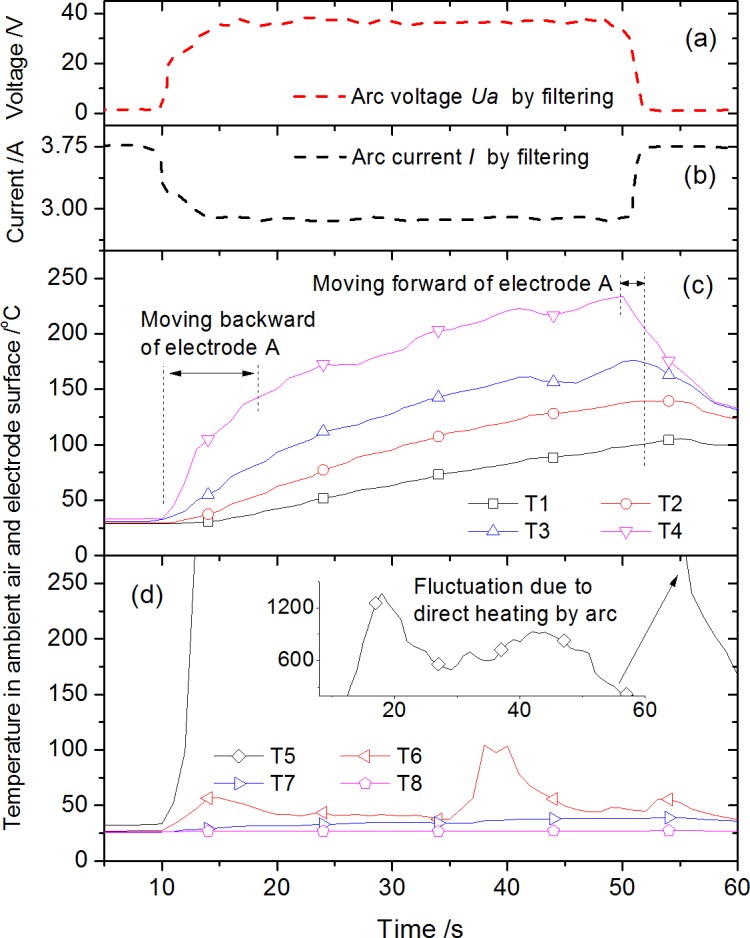
Variation of the characteristic parameters in arc growth: arc voltage (a), arc current (b), temperature along electrode (c) and temperature in ambient air (d) under condition *L* = 2 mm, *U* = 200 V and *R* = 40 Ω.

The arc or arc-fault would lead to changes for both electrical and thermal parameters. Taking the condition of *L* = 2 mm, *U* = 200 V and *R* = 40 Ω as an example, typical variations of the main characteristic parameters measured including arc voltage, arc current, temperature along electrode surface and ambient air temperature are presented in Fig 5. To eliminate the interference by high frequency noise signal in circuit, digital filtering method was adopted for both Fig 5(A) and 5(B). First, since the Volt-Ampere characteristic of arc is somewhat similar to resistor, a step increase trace of arc voltage *U*_*a*_ after the growth of arc could be found in Fig 5(A). *U*_*a*_ maintains almost constant and stable when arc gap reaching to the preset value. Meanwhile, the arc current, equaling to the circuit current *I*, shows a step decrease consequently as shown in Fig 5(B). Second, temperature rises along the electrode surface measured by thermocouple array T1-T4 are shown in Fig 5(C). The surface temperatures by T1-T4 increased gradually mainly due to the heat conduction transfer from arc to electrode, and temperature by T4 (the nearest surface measurement point to arc) would surely increase much faster. As the electrode A was moved towards electrode B at time = 50 s to extinguish the arc for end of test, the temperatures showed decrease tendency subsequently. The temperatures in ambient by T5-T8 are shown in Fig 5(D). Temperature curve of T5 shows an evident fluctuation with maximum value ~1500 K, which is caused by the “direct heating” (say, T5 was contacted with arc directly intermittently due to the arc stream flickering) of arc outer layer with high temperature. T6 was also influenced by the flickering ambient hot gas flow induced by arc stream. With the reduction of convection heat transfer, the ambient air temperature decreased quickly in radial direction as shown by T7-T8.

### Estimation of the arc Joule heating

**Fig 6:**
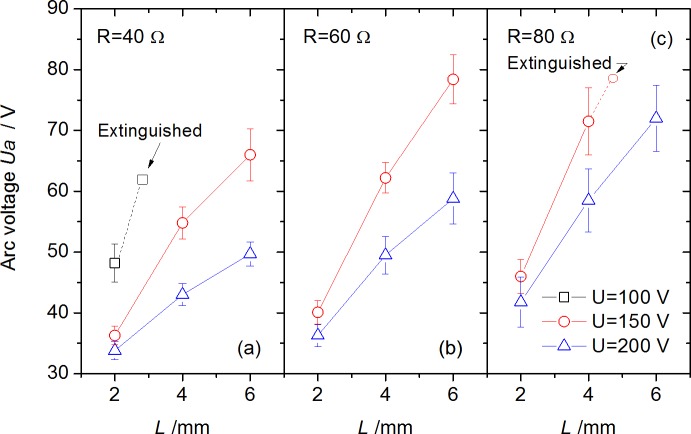
Arc voltage with various arc gap under different circuit voltages and resistances.

**Fig 7:**
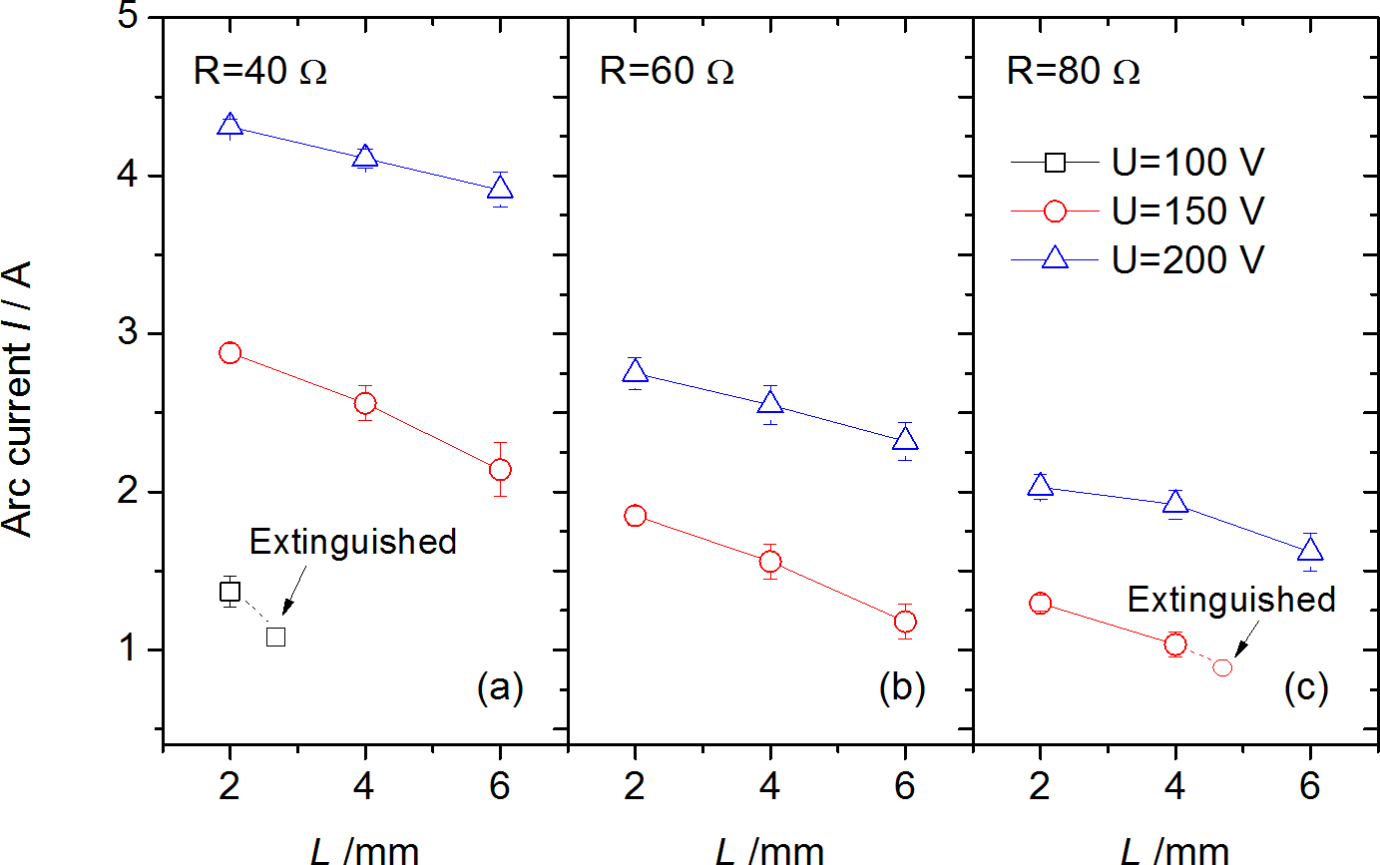
Arc current with various arc gap under different circuit voltages and resistances.

The Joule heating power from arc stream, which is nearly determined by arc voltage *U*_*a*_ and current *I*, is one of the important reasons for electrical fire accidents. The *U*_*a*_ and *I* in arc steady stage for each test are plotted in Fig 6 and Fig 7 respectively.

**Fig 8:**
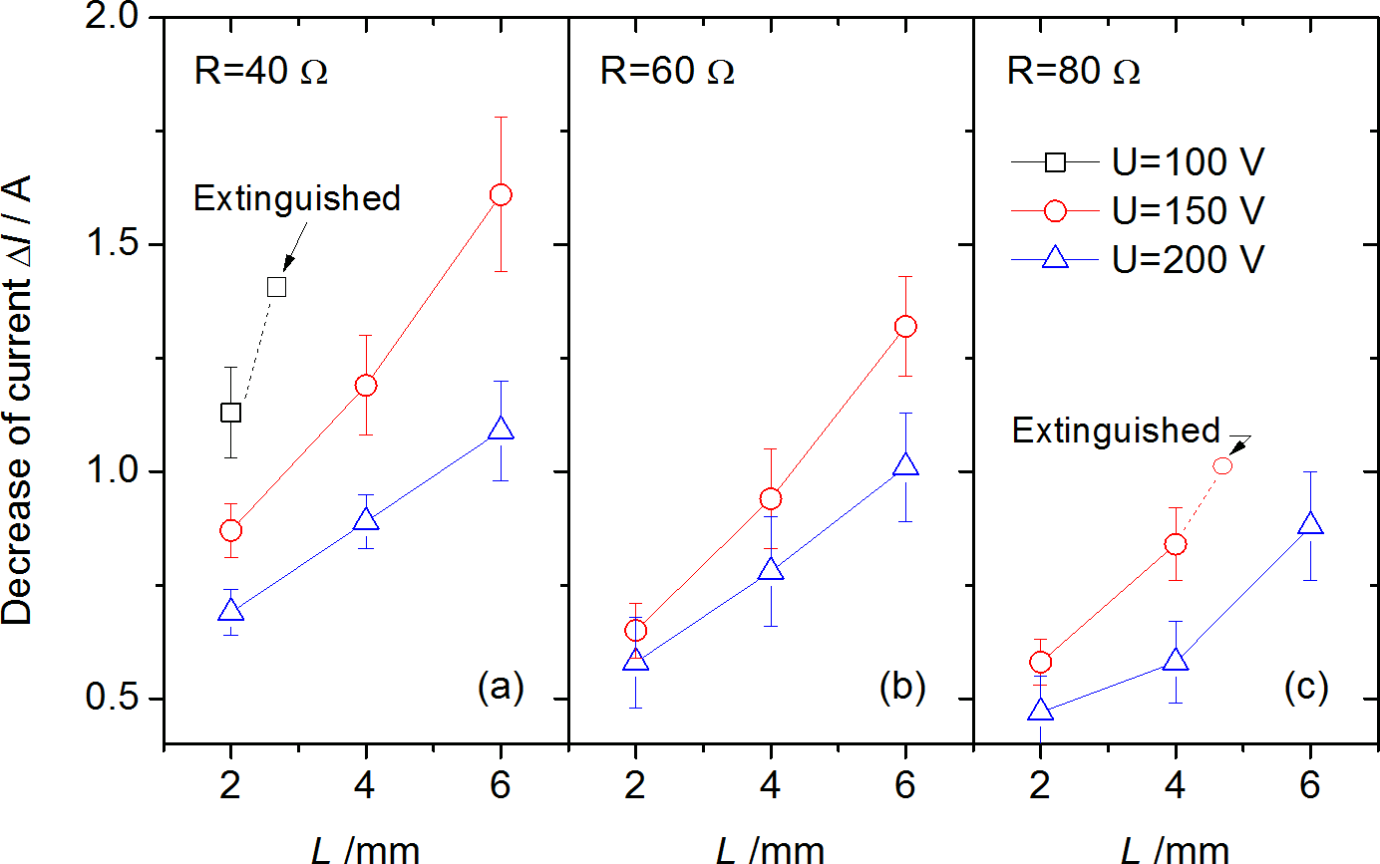
Decrease of Arc current with various arc gap under different circuit voltages and resistances.

**Fig 9:**
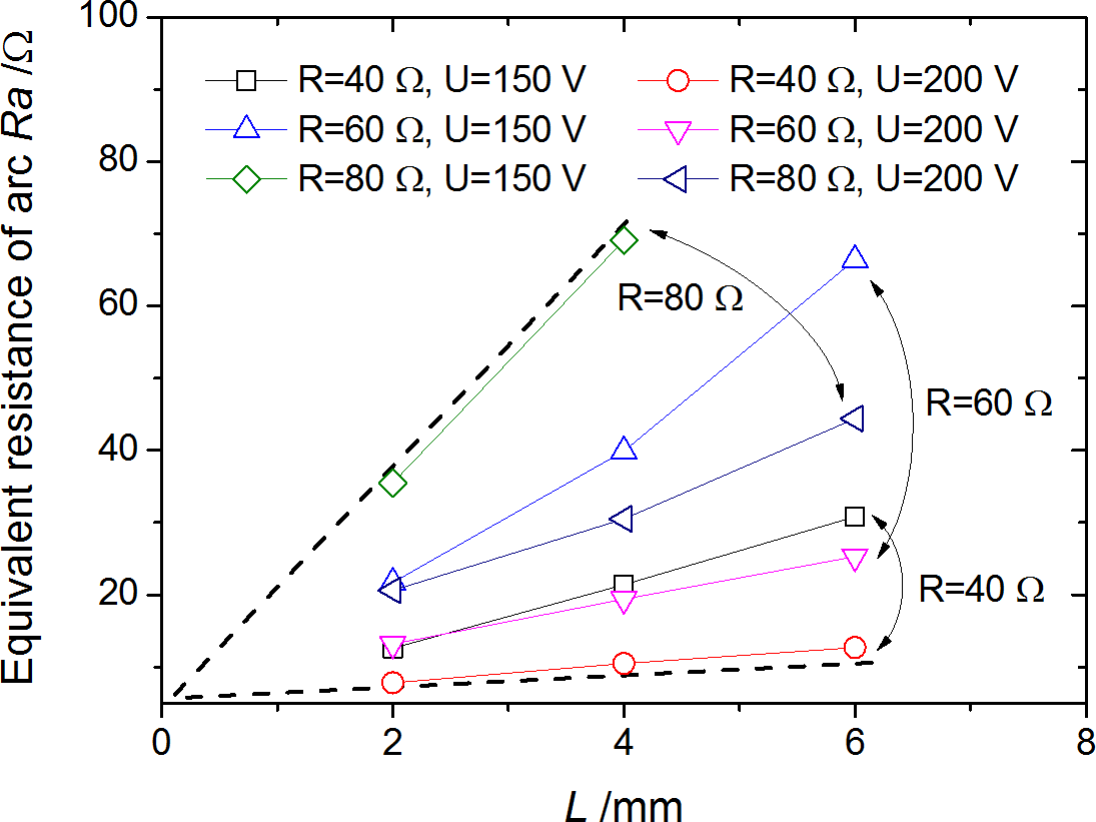
Approximately linear relationship of arc equivalent resistance vs. arc gap.

For a better expression of the influence of arc on current change, Fig 8 gives the decrease of circuit current in detail, defined as Δ*I* = *I*_0_−*I*_*a*_, where *I*_0_ is the initial circuit current without arc occurring, and *I*_*a*_ is the arc current in steady stage measured. These tendencies could be interpreted by a further deduction:

$$\Delta I=\frac{U}{R}-\frac{U-U_{a}}{R}=\frac{U_{a}}{R}$$ (1)

where the relationships of *U*_*a*_ vs. *U* and *U*_*a*_ vs. *R* were already obtained as shown in Fig 6. In fact the results calculated by Eq (1) are closed to the results measured in Fig 8. Moreover, the equivalent resistance of arc, defined as $R_{a}=\frac{U_{a}}{I_{a}}$, is shown in Fig 9. An approximately linear relationship of *R*_*a*_ vs. *L* was found, which was interesting but not surprising by combining the equation $R_{a}=\frac{U_{a}}{I_{0}-\Delta I}=\frac{U_{a}}{U/R-U_{a}/R}=R\cdot \frac{U_{a}}{U-U_{a}}$ and relationships of *U*_*a*_ vs. *R* in Fig 6.

**Fig 10:**
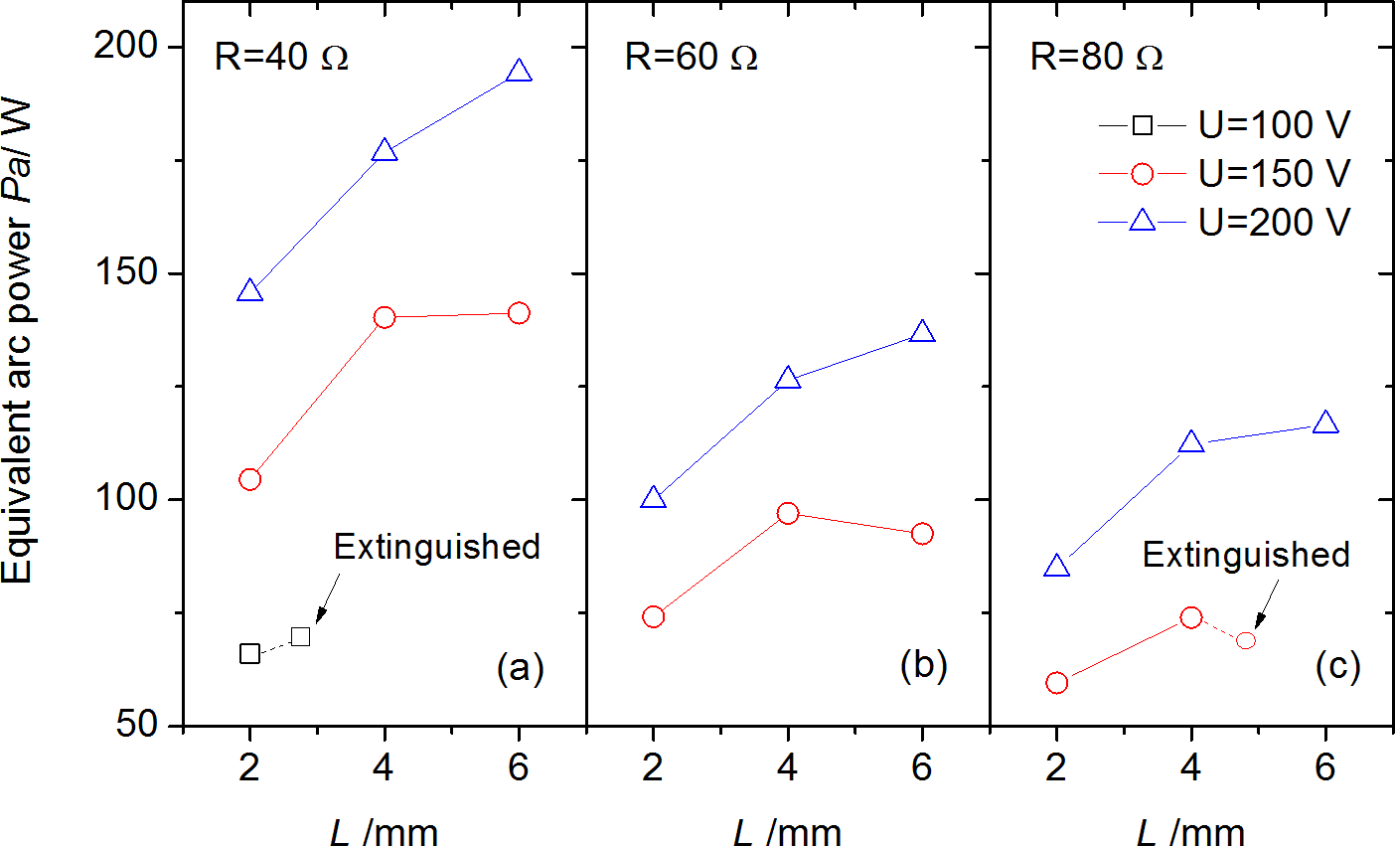
Equivalent arc power vs. arc gap under different circuit voltages and resistances.

Based on the electrical parameters measured above, the equivalent Joule heating power is summarized in Fig 10, by a simplified equation *P*_*a*_ = *U*_*a*_∙*I*_*a*_. It should be noted that with the increasing of arc gap *L*, the equivalent power *P*_*a*_ would have an asymptotic tendency or even decrease tendency (e.g., *U* = 150 V) for some large *L* and small current conditions as shown under *U* = 150 V in Fig 10(B) and 10(C) due to the unstable status before extinguish at arc gap limitation.

### Temperature increase of the electrode surface and ambient air

**Fig 11:**
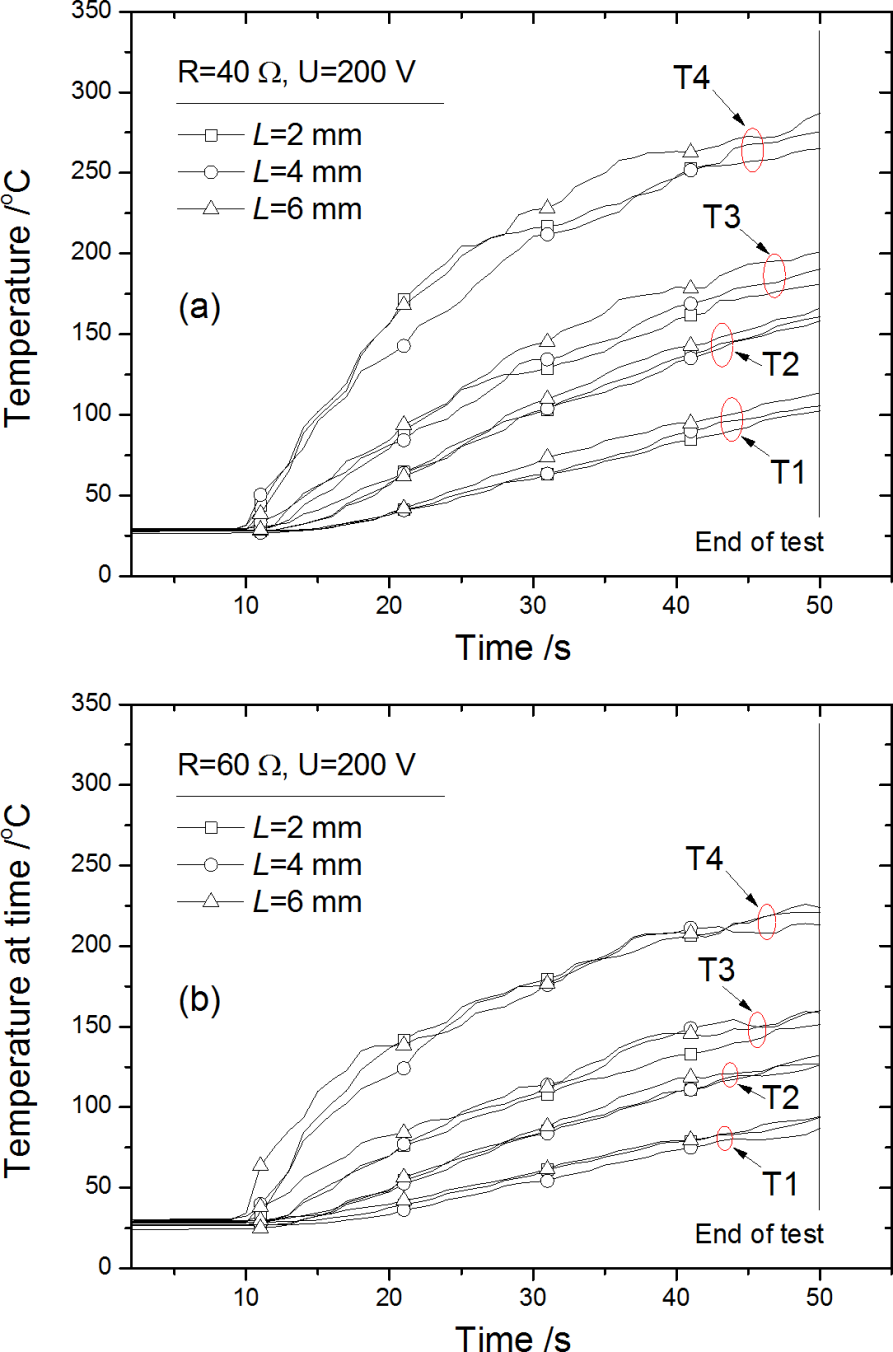
Typical temperature development along electrode surface by T1-T4 for resistance *R* = 40 Ω (a) and 60 Ω (b) with voltage *U* = 200 V.

Temperature rise was driven by the heat release (Joule heating) of arc through conduction, convection and radiation basically. For the electrode surface far from the arc root, the arc gap *L* has a very limited effect on the temperature distribution shown in Fig 11. The explanation for these results is suggested that, First, the conduction heat transferred to the electrode is primarily dependent on the arc temperature of the polar zone, which is not so sensitive to the arc gap. Second, Fig 10 showed that the influence of arc gap on the equivalent power increase is within about 25% for the difference between *L* = 6 mm and *L* = 2 mm (for all tests), and quite a portion of the power should be released to ambient air by convection and radiation from the arc stream in gap, which limits the variation of conduction heat transfer to electrode. Since the same tendency of temperature development in Fig 11, a comparison at a given time would be conducted as below.

**Fig 12:**
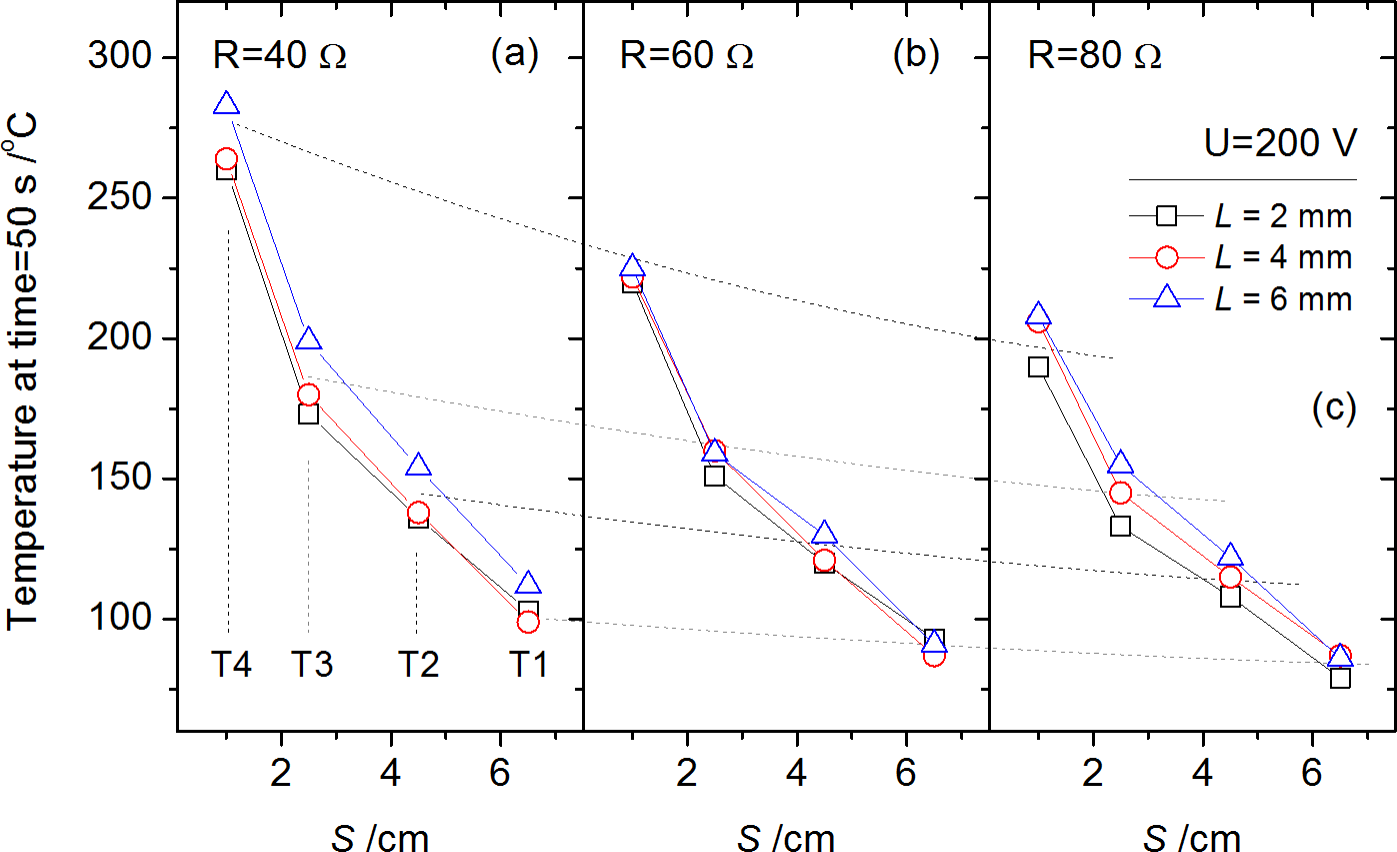
Temperatures along electrode surface by T1-T4 at a given time (50 s) for each test with initial circuit voltage *U* = 200 V.

**Fig 13:**
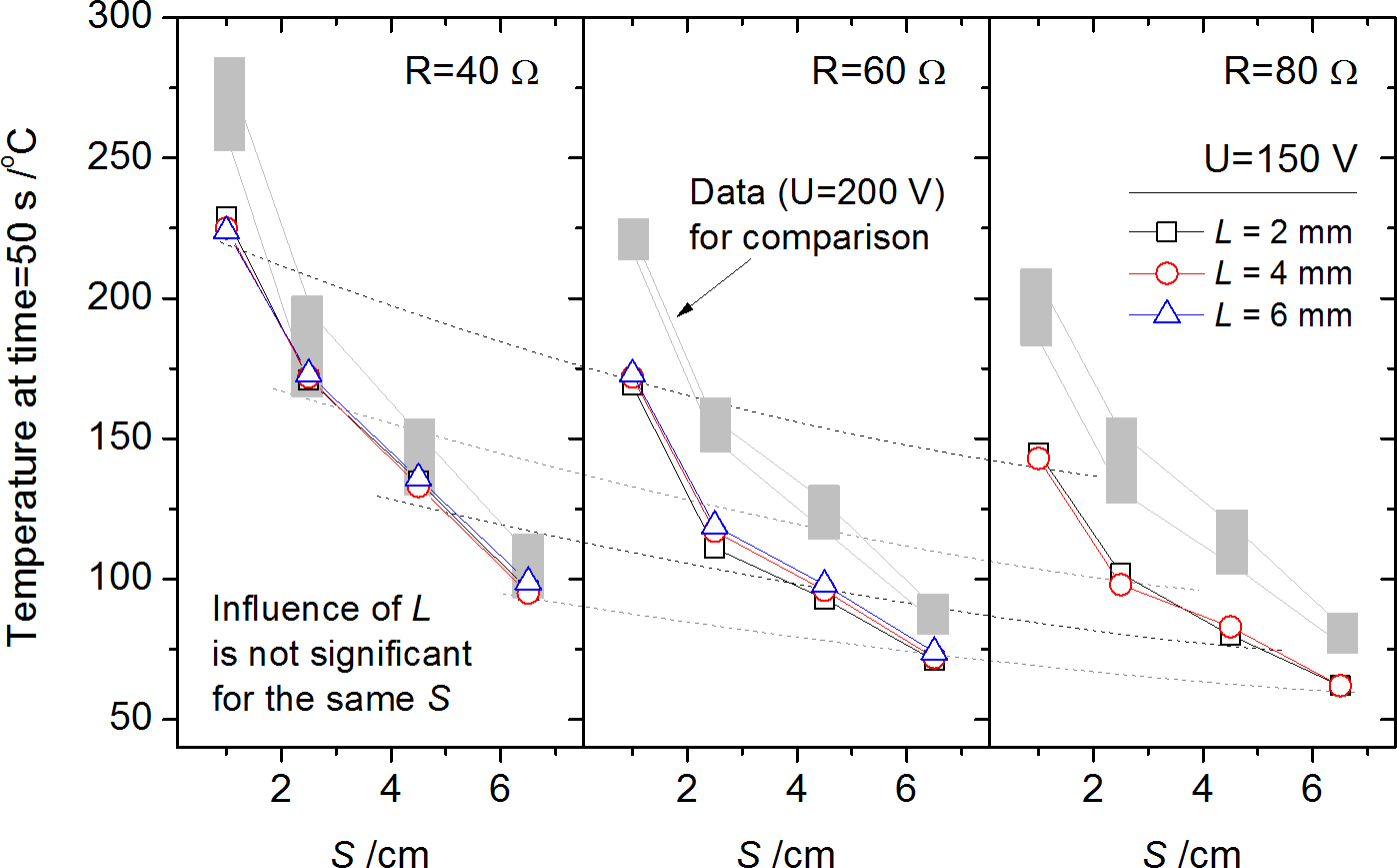
Temperatures along electrode surface by T1-T4 at a given time (50 s) for each test with initial circuit voltage *U* = 150 V.

Considering the extinguishing procedure started at time = 50 s, the temperature distribution along electrode surface at time = 50 s measured by T1-T4 under condition *U* = 200 and 150 V is selected for comparison as in Figs 12 and 13. Temperature rise vs. measuring position *S* shows an approximately exponential decrease. Data of condition *U* = 200 V in Fig 12 is again plotted in Fig 13 for comparison in trend using gray block shapes, which showed the outline of the data in Fig 12. It shows the enlarged voltage *U* would make an entire increase of electrode temperature. The reason is that the increase of equivalent power caused by enlarged voltage *U* (from 150 V to 200 V) is about 30–50%, much larger than the influence of arc gap *L* from 2 mm to 6 mm mentioned before. The influence of *U* on *P*_*a*_ shows increase with the increasing resistance *R*, i.e., the difference of temperature distribution for *U* = 150 V and *U* = 200 V under *R* = 80 Ω condition is larger than *R* = 40 Ω condition as shown in Fig 13. This phenomenon accords well with the arc power differences in Fig 10.

**Fig 14:**
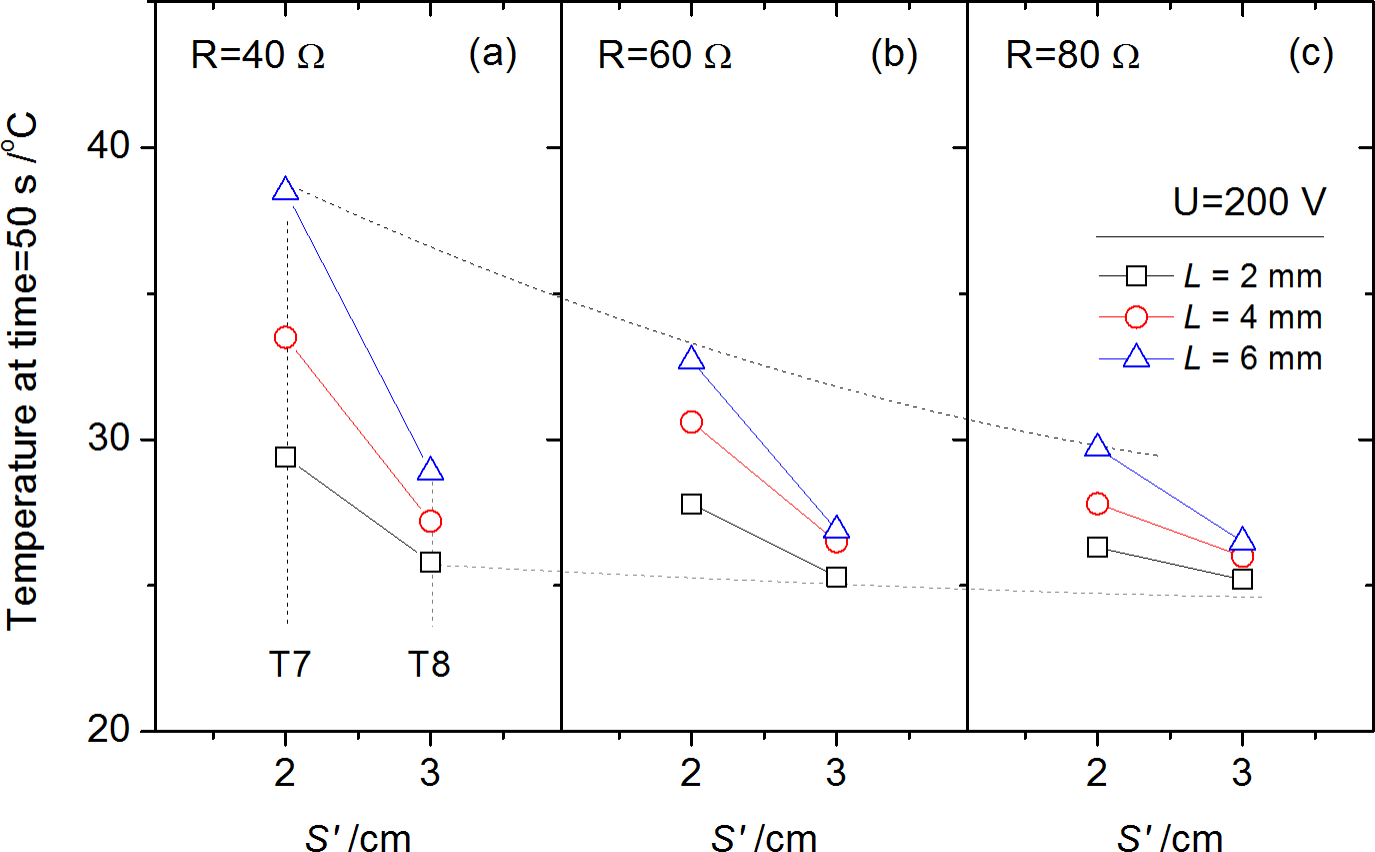
Temperatures of ambient air by T7-T8 at a given time (50 s) for each test with initial circuit voltage *U* = 200 V.

Temperatures of ambient air at the radial direction (time = 50 s) by T7-T8 are shown in Fig 14. Data of T5-T6 are not used considering the disordered fluctuation appeared in Fig 5. The temperature increases by T7 and T8 is relatively small attributed to the distance from arc stream. Different from the results of surface temperature shown in Fig 11, the arc gap effects on ambient temperature gradient in Fig 14 are more significant. It validated the description that a considerable part of heat released from arc stream was in the form of convection and radiation heat, which is proportional to the equivalent power, and is also the dominated heat transfer to the ambient air.

## Conclusions

Experiments on characteristics of a series DC arc-fault including both electrical and thermal parameters were conducted under different conditions based on arc-fault simulator platform. Major conclusions are as follows:

1. For the arc dynamic image characteristics, two zones were observed in discharging process. The inner zone, called the high temperature plasma stream core, emitted light of more blue color with the increasing arc length comparing to the initial white color. The thin outer layer with orange light was shown to be a reaction zone for vapor generated from electrodes, which became prominent with the increased arc power.
2. The arc voltage *U*_*a*_ and current drop Δ*I*, two key parameters to estimate the variation of arc power *P*_*a*_ or Joule heating, showed a positive relation with increasing arc gap *L*, circuit voltage *U* or resistance *R*. While *P*_*a*_ showed a non monotonic trend with *L*. It was caused by the development rule of arc stream, which was firstly enhanced, then unstable, and finally extinguished with the enlarged arc gap.
3. Surface temperature rise of electrode was proposed to be affected by the conduction heat transfer from arc power *P*_*a*_. The influence of arc gap *L* on the electrode temperature increase was not obvious, attributed to the limited increase of *P*_*a*_ and conduction heat transfer with *L*. In addition, heat release to ambient reduced rapidly due to the small arc density and volume, resulted in large temperature gradient in radial direction of arc.

## Supporting information

S1 File Data in Fig 5 for variation of the characteristic parameters. (OPJ)

S2 File Data in Fig 6 for Arc voltage with various arc gap. (OPJ)

S3 File Data in Fig 7 for Arc current with various arc gap. (OPJ)

S4 File Data in Fig 11(A) for temperature under R = 40. (OPJ)

S5 File Data in Fig 11(B) for temperature under R = 60. (OPJ)
